# An Accurate Link Correlation Estimator for Improving Wireless Protocol Performance

**DOI:** 10.3390/s150204273

**Published:** 2015-02-12

**Authors:** Zhiwei Zhao, Xianghua Xu, Wei Dong, Jiajun Bu

**Affiliations:** 1 College of Computer Science, Zhejiang University, #38, Zheda Road, Hangzhou 310027, China; E-Mails: zhaozw@zju.edu.cn (Z.Z.); bjj@zju.edu.cn (J.B.); 2 Zhejiang Provincial Key Lab of Data Storage and Transmission Tech, School of Computer Science, Hangzhou Dianzi University, #1158, Baiyang Block#2, Hangzhou 310027, China; E-Mail: xuxhcs@zju.edu.cn

**Keywords:** sensor networks, protocols, link correlation

## Abstract

Wireless link correlation has shown significant impact on the performance of various sensor network protocols. Many works have been devoted to exploiting link correlation for protocol improvements. However, the effectiveness of these designs heavily relies on the accuracy of link correlation measurement. In this paper, we investigate state-of-the-art link correlation measurement and analyze the limitations of existing works. We then propose a novel lightweight and accurate link correlation estimation (LACE) approach based on the reasoning of link correlation formation. LACE combines both long-term and short-term link behaviors for link correlation estimation. We implement LACE as a stand-alone interface in TinyOS and incorporate it into both routing and flooding protocols. Simulation and testbed results show that LACE: (1) achieves more accurate and lightweight link correlation measurements than the state-of-the-art work; and (2) greatly improves the performance of protocols exploiting link correlation.

## 1. Introduction

Wireless sensor networks (WSNs) are becoming a popular platform for real-world monitoring or controlling systems and applications (e.g., forest monitoring [1], healthcare [2], smart homes [3], green buildings [4], *etc.*). The sensor nodes are equipped with sensors, CPUs and radios with low energy consumptions. They can support long-term and pervasive deployment [5–7]. Routing [8,9] and flooding [10,11] are two important fundamental protocols in WSN-based applications. The routing protocol is used for monitoring: a number of sensors collect (and possibly process) the sensing data of interest and then transmit the data to a sink node (or base station node) in a multi-hop manner. When the data of interest are collected, the flooding protocol is used to perform the controlling process: the sink node transmits the controlling messages to all network nodes to perform certain actions, optimize parameters, *etc.* For example, in the case of smart air-conditioner systems, such as [12], each node first transmits air data (temperature, CO concentration, PM2.5, *etc.*) to the sink node (controlling node) via the opportunistic routing (OR) protocol. Then, the sink node transmits the controlling commands (warming, cooling, aeration, *etc.*) to all nodes *via* flooding to perform certain actions.

Recent studies have observed that link correlation has a strong impact on the performance of both kinds of protocols [13]. We denote link correlation as the correlation of packet receptions/losses on different links from the same sender's broadcast. For example, for the opportunistic routing (OR, which is a popular routing protocol used in WSNs) protocol, when links are positively correlated, there is less spatial diversity to exploit, and the OR performance is likely to degrade; for the flooding protocol, when links are negatively correlated, the broadcast will be less effective, and the retransmission overhead is likely to increase.

Based on this observation, some recent works have proposed to exploit link correlation for improving the performances of OR and flooding. Basalamah *et al.* [14] employ link correlation to estimate the effectiveness of a routing forwarder and select forwarders with a weak outbound link correlation for efficient opportunistic routing (*i.e.*, there is much spatial diversity for this forwarder). Similarly, Wang *et al.* [15] extend a link correlation-aware routing metric that favors good link quality and spatial link diversity (negative link correlation) for efficient routing. Zhu *et al.* [16] use link correlation to reduce the ACK overhead: given two positively correlated links, if the transmission succeeds on one link, the transmission is likely to succeed on the other link, such that we can use one link's ACK to infer the other links' ACKs, avoiding the ACK storm problem. Guo *et al.* [17] use link correlation to construct a tree structure for flooding. The structure favors nodes with strong link quality and link correlation.

However, basically, all of these works heavily rely on the accuracy and efficiency of link correlation measurement/estimation. For example, in [17], if the link correlation is measured inaccurately, the structure nodes (expected to have a strong link correlation) may have a poor link correlation, and the performance will not be improved or may even degrade.

To measure link correlation, the widely-used approach in these works is the beacon-based measurement (BCM). With this approach, each node periodically transmits beacon messages and records the received beacon receptions/losses in a bitmap (denoted as “1” for reception and “0” for loss). Each node then broadcasts its bitmaps to the corresponding beacon sender nodes. After receiving the bitmaps, a node can calculate the correlations between each pair of its outbound links. For example, the link correlation between links *S* → *R*1 and *S* → *R*2 can be calculated as: $c_{R1,R2}^{S}=\frac{N^{CR}}{N_{R2}^{R}}$, where *N^CR^* denotes the number of common packet receptions of two links and $N_{R2}^{R}$ denotes the number of received packets at R2.

With real-world experiments and analysis, however, we observe that the beacon-based approach has the following drawbacks: First, it lacks accuracy. Link correlation is often used to estimate performance metrics (e.g., the expected number of transmissions) of data transmissions. More specifically, link correlation should be essentially the correlation of data packet receptions/losses. However, beacon messages are different from data packets in both packet length and transmission interval, which, as we will discuss in Section 2, are two important factors impacting link correlation. Second, BCM relies only on the historical statistics. It cannot provide timely (short-term) link correlation measurement. For WSN-based applications requiring real-time communications, a timely link correlation measurement is necessary [1,18]. Third, periodically exchanging beacons incur considerable measurement overhead. Before each batch of data packet transmissions, the protocols exchange a large number of beacon messages for measuring the link correlation. These extra transmissions may even balance out the benefits by considering link correlation in the protocols.

To address the above-mentioned problems, in this paper, we propose a lightweight and accurate link correlation estimation approach (LACE). Compared with BCM, LACE has three salient features: First, instead of beacon messages, LACE directly exploits data packet reception/loss traces for measuring the desired data packet-level link correlation. Second, LACE also combines the PHY layer information, the received signal strength indicator (RSSI), to further calibrate the measurement results. Since RSSI indicates the immediate link behaviors, we can extract short-term link correlation with the RSSI trace. By combining both the RSSI-based result and the data reception trace-based statistical result, we can get a timely, yet accurate estimation of the link correlation. Third, LACE uses beacon messages only in the startup session. After that, LACE depends solely on the data reception history and the immediately measured RSSI traces. Considering that RSSI measurements are fast and cheap [19] and that we have almost eliminated the beacon exchanges, the estimation overhead is greatly reduced compared to BCM.

We implement LACE in TinyOS 2.1.2 with TelosB motes as a stand-alone interface. Then, we incorporate LACE into existing link correlation-based OR and flooding protocols to study its impact on end-to-end protocol performance (the number of transmissions and delay). Simulation and testbed experimental results show that LACE: (1) provides more accurate link correlation measurement; (2) greatly reduces the measurement overhead; and (3) that LACE-based protocols outperform their counterparts with BCM.

The contribution of this paper is summarized as follows:

- We demonstrate the inaccuracy of beacon-based measurement and give the reasoning (Section 2).
- We propose a simple model to bridge the PHY layer information (packet length and transmission rate) and packet-level link correlation (Section 3)
- We propose a novel link correlation estimation approach, LACE. LACE combines both link layer and PHY layer information for lightweight and accurate link correlation estimation (Section 3).
- We implement LACE in TinyOS 2.1.2 and incorporate it into existing protocols. Experimental results show that LACE provides more accurate link correlation measurement and that LACE-based protocols outperform existing BCM-based protocols (Section 4).

## 2. Preliminaries and Related Works

### 2.1. Link Correlation Metric and Measurement

Metric: There are several metrics characterizing packet-level link correlation [13,16,17]. Among all of these metrics, conditional packet reception/loss probability (CPRP/CPLP) is the most widely-used, due to its meaningfulness and applicability for estimating upper layer performance, such as the expected number of transmissions (ETX). More specifically, the link correlation-aware opportunistic routing (CAR) [15] and collective flooding (CF) [16] uses CPRP as the link correlation metric, while correlated flooding [17] and CoCo [20] use CPLP as the link correlation metric.

Measurement: Existing works employing beacon messages for measuring link correlation function as follows: Each node periodically transmits beacon messages and records the beacon receptions/losses from other nodes with bitmaps, a “1” denoting a packet reception and a “0” denoting a packet loss. Then, each node transmits the bitmaps to corresponding beacon senders. When a node obtains the bitmaps, it is able to calculate the link correlation (CPRP/CPLP) between its outbound links. For example, with one sender S and two receivers A and B, given two bitmaps indicating the packet receptions on S→A and S→B, “10011” and “11001”, the link correlation can be calculated as the number of common packet receptions divided by packet receptions on S→B, *i.e.*, $c_{A,B}^{S}=\frac{2}{3}$. This means S→A will receive a packet with a probability of 2/3 given that S→B receives a packet.

The above beacon-based link correlation measurement has two main drawbacks, as follows: First and most importantly, it lacks accuracy. Beacon-based measurement uses the beacon receptions to measure the link correlation. As we analyze in Section 2.2, since both the packet length and transmission rate of beacons are different from those of data packets, the fractions of interference captured by beacon-based measurement are different from the interference that has a real impact on data transmissions and the measurement result lacks accuracy. Second, the periodic beacons incur considerable overhead. In [17], beacons are transmitted every 10 s. However, in typical sensor networks, data packets are transmitted every 10 min [1]. The beacons' overhead is unacceptable.

Compared to the above commonly-used approach, LACE: (1) uses data reception bitmaps for link correlation measurement, which is inherently accurate (the elimination of beacons also greatly reduces the measurement overhead); and (2) combines both physical layer parameter and network layer statistics for flexible link correlation estimation (considering both long-term and short-term link behaviors for correlation measurement).

### 2.2. Link Correlation Reasoning and Measurement

Previous works have concluded that link correlation is greatly affected by shadow fading and interference [13,21]. If two nodes have similar signal strengths and interference, they are more likely to receive/lose packets at the same time. As a result, the link correlation between the two links would more likely be strong.

Data transmission rate and packet length: With the above two factors, however, we can still not decide the link correlation. The reason is that the underlying interference and noise are varying, and the data transmissions capture only part of the interference and noise. For example, of two links *A* → *B* and *A* → *C*, when there is an interfering source (e.g., WiFi AP) near *B* and *C*, it is more likely that the two links have similar packet losses and are thus highly correlated. However, the two links may also be independent if the transmissions from *A* happen at the whitespace of WiFi communication. We can see that the interference and noise captured by the packet transmissions (the interference and noise covered by grey parts) essentially decide the link correlation.

To decide the interference and noise fractions captured by the packet transmissions, clearly, the packet length and transmission rate are the two important factors (it is worth noting that in many WSN-based applications, data packets are transmitted with fixed data rate [1]).

**Figure 1.**
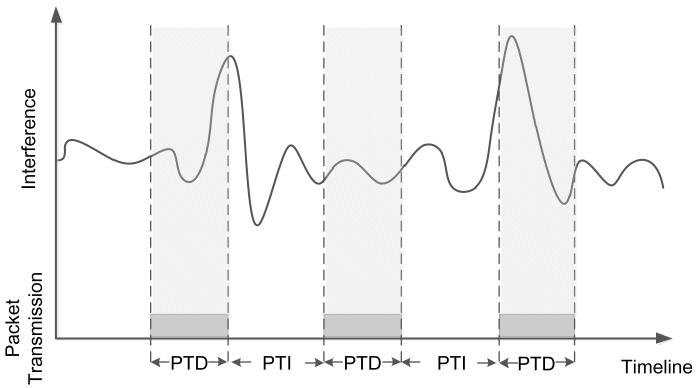
An example to illustrate the impacting factors of link correlation. PTD, packet transmission duration; PTI, packet transmission interval.

To more clearly illustrate the impact of the two factors, we use a simple example with one sender and two nearly-placed receivers. Nearly-placed receivers have similar signal strength, noise and interference. The sender keeps broadcasting packets, and we study the link correlation between the two links. As depicted in Figure 1, the x-axis denotes time, and the y-axis denotes the environmental interference. The grey blocks denote packet transmissions. PTD denotes the packet transmission duration, which is determined by the packet length. PTI means packet transmission interval. We can see that the three packets capture different fractions of interference, resulting in certain link correlation values. As a result, Packets 1 and 3 are more likely to be lost at the receivers, while Packet 2 is more likely to be independently received/lost at different receivers. The reason is that interference during Packet 2's transmission is low, while the interference during Packets 1 and 3 is high. Clearly, different PTIs and PTDs will lead to different captured interference and noise, resulting in different link correlations.

Beacon-based correlation measurement (BCM) has two main drawbacks in capturing the link correlation (*i.e.*, the data packet reception/loss correlation). First, from the above observation, beacons have different packet lengths and transmission rates than data packets, capturing different interference patterns with data packet transmissions. Thus, interference that has a large impact on data packet reception/loss correlation may not be captured by the beacons. Second, even the captured interference is similar, since the beacons' packet lengths are different from the data packets, the reception of beacons and data packets will be different. Existing works have concluded that with the same signal-to-interference and noise ratio (SINR), short packets are more likely to be correctly received [22]. Therefore, a data packet loss may be misestimated as a reception using a beacon message, which further yields errors for estimating data packet reception/loss correlations.

Statistical approach: A simple improving approach is to simply use data receptions/losses for measuring link correlation, in which the link correlation is calculated based on the historical traces of packet receptions and losses.

However, since link correlation is often used for routing decisions, the desired link correlation should combine long-term stable behavior, as well as short-term behavior. If there are large batches of packets to transmit, we may require long-term link correlation (the historical data plane link correlation is more meaningful); on the other hand, if we would like to transmit only one or a small number of packets, short-term link correlation (derived from RSSI and noise readings) is preferred.

### 2.3. Protocols Exploiting Link Correlation

We summarize recent works aiming at improving the protocol performance of opportunistic routing and flooding.

Opportunistic routing: The key idea of opportunistic routing is to exploit spatial diversity to improve the OR efficiency, *i.e.*, a forwarder broadcasts a packet, and any relay nodes that overhear the packet can become the next forwarders. Compared to traditional unicast routing, it can reduce the number of retransmissions.

Basalamah *et al.* [14] identify that link correlation indeed has a large impact on the spatial diversity. From the same sender, if the outbound links are weakly correlated, each receiver has different received packets, and there is much spatial diversity. The key insight is that negative correlated links are beneficial to opportunistic routing. The reason is that with negative link correlation, if a receiver cannot receive the packet, it is more likely that another receiver can receive that packet. Then, we can infer that with negative correlated links, when a packet is broadcast, at least one receiver can receive the packet, and retransmissions are avoided. By selecting forwarders with negative correlated links, the protocol can effectively exploit the spatial diversity in the network and reduce transmission and delay overhead. Compared to negative correlated links, independent links (with weak or no link correlation) are less beneficial.

Wang *et al.* [15] extend the work in [14] by formally designing a link correlation-aware routing metric (expected number of transmissions, ETX), which considers both link quality and link correlation. Moreover, it designs an efficient prioritization approach to prioritize the transmissions from high metric value forwarders. By efficiently selecting the weakly correlated links with high link qualities, the routing efficiency is improved.

Flooding: Traditional flooding is simple. The sink node broadcasts the flooding packets, and nodes that received the packets reply ACKs and then re-broadcast the packets. This process goes recursively until all network nodes receive the packets.

Zhu *et al.* [16] employ link correlation for efficient flooding. The proposed protocol, CF (collective flooding), improves the flooding performance compared to previous work. CF uses link correlation to infer the probability of a packet's reception at different receivers. Based on this knowledge, CF calculates the impacts of different forwarders and selects the best forwarder with the largest impact.

Guo *et al.* [17] exploit link correlations for efficient flooding in low duty cycle sensor networks. Nodes with high correlated links are grouped together in order to reduce the number of ACKs. The grouping of nodes, however, requires each node to perform a time-consuming k-means algorithm, which incurs a large computational overhead on resource-constrained sensor nodes. Iftekharul *et al.* [23] extend Zhu *et al.'s* design [16] for multiple packet scenarios by employing rateless codes, which improve the performance in terms of transmissions.

Wang *et al.* [21] consider link correlation in the clustering of network nodes (CorLayer). CorLayer models the relationship between link correlation and the expected number of transmissions (ETX) and then selects nodes with small ETXs to form clusters. Then, by connecting all clusters, the underlying structure for efficient flooding is constructed.

Zhao *et al.* [20] employ link correlation for dissemination (multi-message reliable flooding). They use link correlation, as well as link quality to model the ETX of each potential sender during the dissemination process, such that strong link correlation neighborhoods in the network are selected for data forwarding. After that, many optimizations on the protocols are done to further reduce the transmission overhead and energy consumption.

The above-mentioned protocols rely on the beacon-based link correlation measurement (BCM). The inaccuracy of BCM may balance out the benefits of considering link correlation. Moreover, the measurement itself incurs considerable overhead. Our proposed LACE approach can be employed by all of these works, providing more accurate and lightweight link correlation estimation, further improving the protocol performances.

## 3. Main Design

In this section, we give the main design of LACE.

### 3.1. Overview

Our key idea comes from the observation that data and beacon capture totally different interference and noise patterns, and the statistical approach lacks short-term estimation.

**Figure 2.**
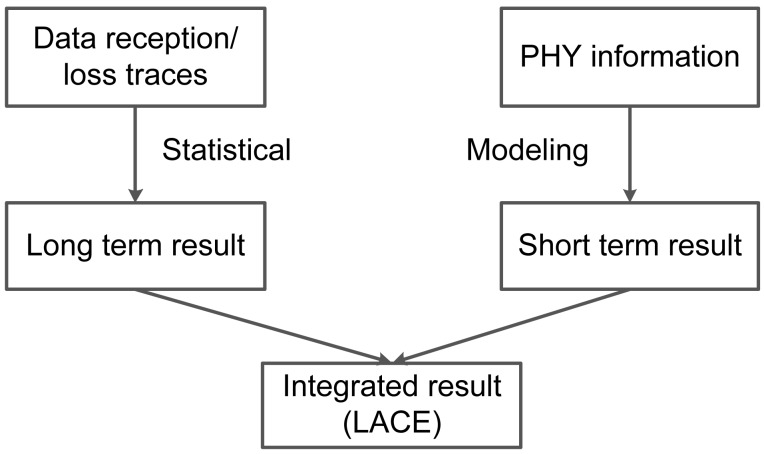
Lightweight and accurate link correlation estimation (LACE) overview.

At a high level, the proposed link correlation estimation has two main parts, as shown in Figure 2: long-term estimation and short-term estimation. Long-term estimation comes from the statistics of historical packet receptions and losses. Short-term estimation comes from the modeled relationship between SINR values and expected link correlation (Section 3.2). Then, we integrate the long-term and short-term link correlation with a weighting factor *α*. When used for large batches of packet transmissions, we use a large *α* value to represent long-term link correlation; when used for a single packet or a small number of packet transmissions, we use a small *α* value to represent short-term link correlation. We first give the notations used in this section in Table 1.

### 3.2. LACE Measurement

We use a simple example as follows to illustrate the workflow of LACE. *S* periodically transmits data packets to its downstream nodes, *A* and *B*. When receiving the data packets, *A* and *B* record the packet receptions/losses from *S* in bitmaps, with a “1” denoting a packet reception and a “0” denoting a packet loss. At the same time, *A* and *B* record the noise and interference, as well as the received signal strength of *S*'s transmissions. Furthermore, periodically, receivers *A* and *B* reply the bitmaps, the noise and interference traces and the signal power traces back to node *S*, such that *S* is able to calculate link correlation $C_{A,B}^{S}$ as follows:

(1) $$c_{A,B}^{S}=\alpha \cdot ch_{A,B}^{S}+\left(1-\alpha \right)\cdot ci_{A,B}^{S}$$

where $ch_{A,B}^{S}$ is the historical correlation value (estimated from the bitmaps), $ci_{A,B}^{S}$ is the instant correlation value (estimated from the PHY layer information) and *α* is a weighting factor. When *α* is large, long-term estimation is weighted more, while when *α* is small, short-term estimation is weighted more.

Long-term estimation: With the bitmaps from *A* and *B, S* can calculate $ch_{A,B}^{S}$ as:

(2) $$ch_{A,B}^{S}=\frac{\sum_{i=1}^{n}\left(b_{A}\left[i\right]\&b_{B}\left[i\right]\right)}{\sum_{i=1}^{n}b_{B}\left[i\right]}$$

where & denotes the bitwise AND operation and *b_A_*[*i*] denotes the *i*-th bit in A's bitmap *b_A_*. There are different link correlation metrics [13,16,17], and the above equation essentially calculates the metric of the conditional packet reception probability. Different upper layer protocols may require different correlation metrics. It is worth noting that we can support all of the metrics for calculating packet reception/loss correlations. For example, the metric of conditional packet loss probability can be calculated as: $ch_{A,B}^{S}=\frac{\sum_{i=1}^{n}\left(\neg b_{A}\left[i\right]\&\neg b_{B}\left[i\right]\right)}{\sum_{i=1}^{n}\neg b_{B}\left[i\right]}$ where ¬ is the bitwise negation operation.

The calculation is similar to the BCM approach. The difference with BCM is that: (1) the bitmaps are recorded for data packet receptions/losses, which are representative of data plane link correlation (as discussed in Section 2, it is the true reflection of the correlations of data packet receptions and losses); (2) the use of data receptions and losses eliminates the need for extra periodic beacon messages, which greatly reduces the transmission overhead; and (3) it is also worth noting that there are also cons to our approach. Our approach can yield accurate link correlation estimation only when there are periodic data packets. When there are few or even no data packet transmissions, our approach cannot work well, since there are no data traces.

Short-term estimation: Next, we use RSSI to infer the instant link quality of the two links and further estimate the short-term link correlation. When estimating the short-term link correlation with *m* packet transmissions, we can calculate $ci_{A,B}^{S}$ as:

(3) $$ci_{A,B}^{S}=\sum_{k=0}^{m}ct_{A,B}^{S}\left(k\right)\cdot R\left(n_{r}=k\right)$$

where $ct_{A,B}^{S}\left(k\right)$ is the link correlation value with *k* common packet receptions among *m* packets, and *R*(*n_r_* = *k*) denotes the probability with *k* common packet receptions. $ct_{A,B}^{S}\left(k\right)$ is given by:

(4) $$ct_{A,B}^{S}(k)=\frac{k}{\overset{m}{\underset{j=1}{\text{∑}}}p_{B}^{S}\left(j\right)}$$

where *k* denotes the number of common packet receptions and $p_{B}^{S}\left(j\right)$denotes the *j*-th packet reception probability. The denominator calculates the expected number of receptions by node B.

*R*(*n_r_* = *k*) is calculated as the average probability with *k* common packet receptions on both links:

(5) $$R\left(n_{r}=k\right)=\frac{\underset{\forall S_{k}\in S_{m}}{\text{∑}}\left(\overset{k}{\underset{j=1}{\text{∏}}}p_{A}^{S}\left(j\right)\cdot p_{B}^{S}\left(j\right)\overset{m}{\underset{j=k+1}{\text{∏}}}\left(1-p_{A}^{S}\left(j\right)\cdot p_{B}^{S}\left(j\right)\right)\right)}{\left(\begin{array}{c} m\\ k \end{array}\right)}$$

where *S_k_* denotes the set of *k* common packet receptions, *S_m_* denotes the set of *m* packets and $p_{B}^{S}(k)$ denotes the *k*-th packet reception probability. $\overset{k}{\underset{j=1}{\text{∏}}}p_{A}^{S}\left(j\right)$ denotes the probability of *k* common packet receptions. $\overset{m}{\underset{j=k+1}{\text{∏}}}\left(1-p_{A}^{S}\left(j\right)\cdot p_{B}^{S}\left(j\right)\right)$ denotes the probability that the other *m* − *k* packets have no common packet receptions. For one packet transmission, the probability that both receivers receive the packet is simply the multiplication of the two packet reception rates of the two links. The reason is that from the PHY layer, a packet's reception depends only on the SINR (signal-to-interference and noise ratio). When two links have a high correlation, this means they have high fractions of common receptions. However, the receptions on one link do not depend on the other link. Therefore, a packet's receptions on two links are independent of each other. There are $\left(\begin{array}{c} m\\ k \end{array}\right)$ different cases with *k* common packet receptions. Therefore, in total, *R*(*n_r_* = *k*) is the expected probability with *k* common packet receptions.

The above calculation relies on the packet reception probability ( $p_{A}^{S}$ and $p_{B}^{S}$). $p_{A}^{S}\left(i\right)$ is estimated from the SINR value for packet *i* and is given according to [24]:

(6) $$p_{A}^{S}\left(i\right)=\prod_{j=1}^{2l}\left(1-es_{A}^{S}\left(i,j\right)\right)$$

The PHY layer is based on direct sequence spread spectrum (DSSS) technology employing offset quadrature phase-shift keying (O-QPSK) modulation. One byte is translated into two symbols, and one symbol is translated into 32 chip-long pseudo-random noise sequences [24]. Then, $es_{A}^{S}\left(i\right)$ is given by:

(7) $$es_{R1}^{S}\left(i,j\right)=\sum_{n=1}^{32}\left(\begin{array}{c} 32\\ n \end{array}\right){\left(ec_{R1}^{S}\left(i,j\right)\right)}^{n}{\left(1-ec_{R1}^{S}\left(i,j\right)\right)}^{32-n}\times P_{\text{symerr}}\left(n\right)$$

where $ec_{A}^{S}\left(i,j\right)$ is the chip error rate for symbol *j* in packet *i*, supposing the chip error rates are identical to the symbols. $ec_{A}^{S}\left(i\right)$ is given by:

(8) $$ec_{R1}^{S}\left(i,j\right)=\frac{1}{2}\left(1-\sqrt{\frac{Sr_{R1}^{S}\left(i,j\right)}{1+Sr_{R1}^{S}\left(i,j\right)}}\right)$$

where $Sr_{R1}^{S}\left(i,j\right)$ denotes the *j*-th SINR sample (identical to a symbol) within packet *i*.

With the above equations, we are able to estimate $ci_{A}^{S}$.

### 3.3. Practical Issues

To implement LACE on sensor nodes, we have several practical issues to tackle.

Reducing complexity: According to the above equations, we can see that to calculate the short-term link correlation, the computational complexity is *O*(2*^m^*), which is high for sensor nodes. Apparently, there is a tradeoff between the accuracy and the complexity. To reduce the overhead, we do not calculate the average for all samples. Instead, we generate several bitmaps according to the *m* packet reception ratios (derived from Equation (6)) and calculate the average value of the generated bitmaps. We introduce a *δ* factor, which is the number of bitmap generations to calculate in Equation (5). Intuitively, when *δ* is large, more bitmaps are generated for calculation; the result will be more accurate, but the overhead will increase. Otherwise, the result will be less accurate, and the overhead will decrease. Therefore, for resource-constrained platforms, delta should be set small; for more powerful devices, delta should be set large. For example, when *δ* = 1, *R*(*n_r_* = *k*) is calculated as:

(9) $$R\left(n_{r}=k\right)=\prod_{j=1}^{k}p_{A}^{S}\left(j\right)\cdot p_{B}^{S}\left(j\right)\prod_{j=k+1}^{m}\left(1-p_{A}^{S}(j)\cdot p_{B}^{S}\left(j\right)\right)$$

for randomly selected *k* common packet receptions. Then, the calculation complexity can be reduced to *O*(*m*^2^), which is much more practical for resource-constrained sensor nodes.

To find an appropriate *δ* value, we conduct multiple experiments with varying *δ* values on the TelosB platform [25]. Our experimental results show that *δ* = 5 achieves accuracy over 70% with only a 1.506-ms delay. For more powerful devices, such as iMote [26], or in delay-tolerant application scenarios, *δ* can be set larger to achieve more accurate estimation.

**Figure 3.**
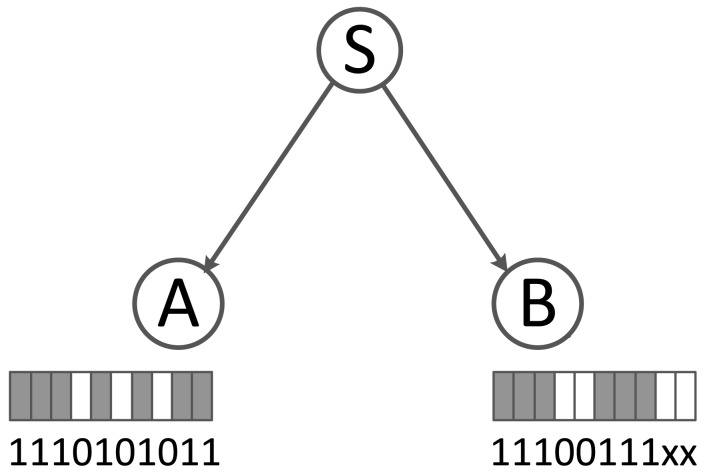
An example for bitmap aligning.

Bitmap issues: As described above, the bitmaps are recorded at receivers and returned to the senders. There are two key issues: First, bitmaps at different receivers should be aligned with the same time offset. We use 32-bit length bitmaps to record the latest 32 received/lost data packets. However, the lost packet is indirectly identified (using the sequence number difference), *i.e.*, when a receiver loses a packet, it can identify this packet loss only when it receives a new packet in the future. As a result, at the time when a node loses a packet, its bitmaps are not updated until the next packet reception. Further, bitmaps of different receivers may record different sets of packet transmissions, and the calculated link correlation would be meaningless (as shown in the example of Figure 3).

For example, sender S transmits 10 packets; node A receives 1,2,3,5,7,9,10, and node B receives 1,2,3,6,7,8. If we set the bitmap length as five and collect the bitmaps after the time of the 10th packet transmission, the bitmaps of A and B will be 01011 and 00111. Clearly, node B's bitmap does not record the latest five packets (6–10); instead, it records Packets 4–8. The reason is that it is unaware of Packet 9 and 10's transmissions.

To solve this inconsistency, we add a initial offset at the beginning of each bitmap to indicate the start position of the bitmap. With the offset, the sender can align the bitmaps and calculate the link correlation. We use the same example as the above to illustrate how this works: When node A and B prepare to return the bitmaps, they add the start positions. Then, S receives two vectors, 6|01011 and 4|00111. Now, S uses the common parts of the bitmaps (010 and 111 for Packets 6–8, respectively) to calculate link correlation *ch* according to Equation (2).

The second issue is the length of bitmaps: when the bitmap is long, it can represents more long-term link behavior, but requires more memory overhead. Considering that the TelosB platform has only 10 KB RAM and that the packet payload length is no longer than 114 bytes, we set the length of bitmaps according to the neighbor size: When a node has many neighbors, it uses short bitmaps to save memory/transmission overhead. When a node has a small number of neighbors, it uses large bitmaps for more accurate estimation. In our experiment, we use 32-bit length bitmaps to record the packet receptions.

### 3.4. Tuning α

As discussed above, we use a weighting parameter *α* to calculate the integrated correlation metric. When *α* is large, the history correlation is estimated with more weight; When *α* is small, the instant correlation is estimated with more weight. If the packet is received/lost at both receivers, we mark the ground truth of link correlation as 1; if the packet is received at only one receiver, we mark the ground truth as 0.

We record the ground truth and adaptively tune *α* for more accurate link correlation estimation. The intuition is that the correlation value close to the ground truth should be more weighted: If the ground truth of packet link correlation is 1, we increase the weight of max (*ch, ci*). If the ground truth of packet link correlation is 0, we increase the weight of min (*ch, ci*). For example, with *ch* = 0.9, *ci* = 0.5 and *α* = 0.5. If the received packets are the same, which means the instant correlation is 1 for the packet, we increase *α* by *inc*, since the *ch* value is closer to 1. Note that the increased value *inc* can also be tuned: *α* with a large *inc* is more sensitive to the estimation results, while *α* with a small *inc* is more accurate. Finding a theoretical optimal *α* will be studied in future works.

## 4. Evaluation

Before presenting the evaluation results, we first show the benefits of considering link correlation in flooding and opportunistic routing, using a simple example with one sender and two receivers. The packet reception rates on both links are 0.5. (1) For flooding, the sender tends to deliver a number of packets (say, 10 packets) to the two receivers. After the first round of transmission, both links have lost five packets. With positively correlated links, the five lost packets on each link will be the same, and the sender needs to retransmit only five packets. However, with negatively correlated links, the five lost packets on each link will be different, and the sender needs to retransmit 10 packets (five packets for each receiver). We can see that, if we select the senders with positive correlation for flooding, the transmissions will be saved [17]; (2) For opportunistic routing, the sender tends to deliver a packet to at least one node of its receivers. With one packet transmission, if the links are positively correlated, there is a 50% probability that both links lose the packet. However, if the links are negatively correlated, *i.e.*, when a receiver loses a packet, the other receives the packet, we can infer that at least one node can receive the packet. Thus, if we select the senders with negative correlated links for opportunistic routing, the performance will be improved [15].

We can infer that if link correlation is inaccurately measured, the above benefits will degrade. Therefore, by improving the estimation accuracy, LACE could be used for further improving the protocol performance of flooding and opportunistic routing.

To study the effect of LACE on protocol performance, we implement a stand-alone interface of LACE in TinyOS on TelosB motes. We first test the accuracy of link correlation estimation in TOSSIMsimulation with a 10 × 10 network (the “Meyer-heavy” noise trace is used and the gain of each link is randomly set in range of [−95,−70] dbm). Next we incorporate LACE into existing network layer protocols (a link correlation-aware opportunistic routing [15] and a reliable multi-message flooding (dissemination) protocol (CoCo) [17]) to study the performance improvements. Our 4 × 10 testbed is used for real motes' evaluation.

### 4.1. The Accuracy of LACE

To study the estimation accuracy of LACE, we use the link correlation calculated with actual data traces as the ground truth: If the two links receive/lose a packet at the same time, the instant correlation is 1. Otherwise, the link correlation is 0. We use LACE to measure the link correlation before each packet transmission and study its accuracy compared to the above ground truth. For example, if the estimated link correlation is 0.8 and the packet is received by both links, the accuracy is 0.8; if the estimated link correlation is 0.8 and the packet is not received by both links (at least one receiver loses the packet), the accuracy is 1 − 0.8 = 0.2.

**Figure 4.**
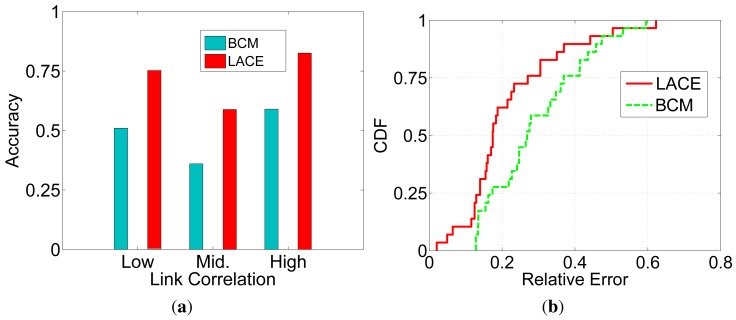
Accuracy of link correlation estimation. (**a**) Average estimation accuracy; (**b**) Variability of estimation errors.

We conduct the experiment 100 times and then calculate the average accuracy for all packet transmissions. Figure 4a depicts the estimation accuracy of LACE. We can see that for low and high link correlation, the LACE accuracy is high, while for intermediate link correlation links, the LACE accuracy is relatively low. This is due to the conditional probability-based link correlation metric having a PRR (packet reception ratio) bias: when the two links have both low/high PRR, the probability that they receive the packet at the same time will also be low/high. As a result, when links are high or low, the estimated link correlation is more accurate. For intermediate links (PRR around 0.5), the estimated *ci* will be around 0.25. However, the actual link correlation with two 0.5 links can be any value from 0∼1. The error is more likely to be large.

The reason for the above bias is the use of the average or the lack of a statistical distribution for link correlation. As discussed in Section 2.2 and in [21], the link correlation variation depends on the background noise and interference patterns. Under different environments, the interference patterns will be different. Thus, we can infer that the link correlation distributions will also be different with different environments. We expect that with a given distribution, the estimation accuracy can be further improved. We would like to study the model and distribution of link correlation in our future works.

LACE is more accurate than beacon-based link correlation estimation (BCM). The reason is that LACE extracts the link correlation directly from the data packet receptions. Due to the difference of data and beacon packets, the beacon-based methods suffer from large estimation errors.

Figure 4b shows the cumulative distribution function (cdf) of the estimation accuracy of LACE and BCM. We can see that when using LACE, about 60% of the estimations have errors under 0.2. While using BCM, only about 25% of the estimations have errors under 0.2. The reason is two-fold: (1) LACE directly uses data packet receptions/losses, which are more likely to capture the noise and interference patterns experienced by periodic packet transmissions; and (2) LACE uses short-term PHY layer information for calibration.

### 4.2. Testbed Results of Protocol Performance

In this section, we study the protocol performance of opportunistic routing (CAOR [15]) and flooding (CoCo [17]) with LACE.

**Figure 5.**
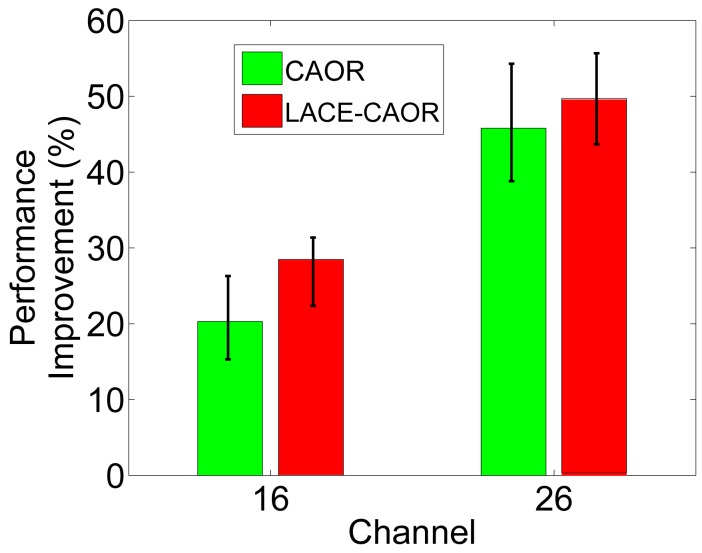
Delay performance evaluation with CAOR [15].

Figure 5 shows the delay comparison of LACE-CAOR and the original CAOR with BCM. The delay here denotes the time from the transmission of a packet to the reception of the packet at the destination node. We can see that LACE-CAOR's routing delay is less than CAOR. Though the same routing strategies are used in CAOR and LACE-CAOR, the measured/estimated link correlation is different. With a more accurate estimation, LACE-CAOR is able to find network neighborhoods with much spatial diversity, while CAOR's routing decisions actually find neighborhoods with less spatial diversity. More specifically, the performance improvement is larger in Channel 16 than in Channel 26. The reason is that Channel 16 is has more severe interference from WiFi traffic and has a stronger link correlation than Channel 26. There are inherently fewer neighborhoods with a weak link correlation in Channel 16 than in Channel 26. As a result, the identification of weak link correlation areas can contribute more to the end-to-end delay performance in Channel 16. On the other hand, most neighborhoods in Channel 26 have weak link correlations, and the BCM-based method can also find a weak link correlation neighborhood. The performance improvement then becomes less.

**Figure 6.**
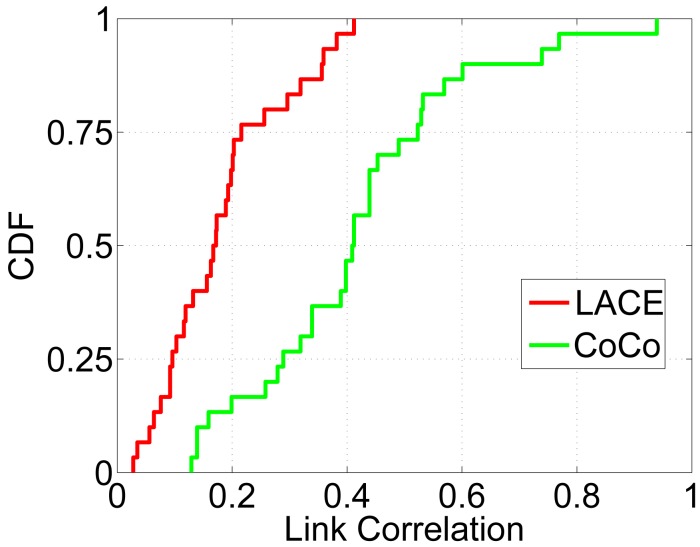
Estimation accuracy with CoCo [20].

**Figure 7.**
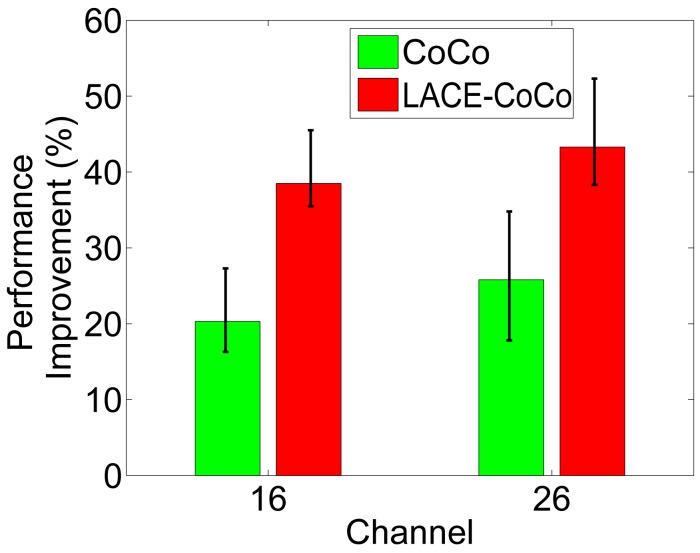
Performance evaluation with CoCo [20].

Figures 6 and 7 evaluate the performance of CoCo and LACE-CoCo. Figure 6 shows the measurement/estimation accuracy of LACE and BCM. We can see that: (1) as expected, LACE is more accurate than BCM; and (2) the accuracy difference is much larger than that in Figure 4b. The reason is that LACE-CoCo aims to disseminate multiple packets, which increases the number of samples for calculating long-term link correlation (while in CAOR, only one packet is transmitted at a time). Since there are more historical traces, the *ch* part can be estimated more accurately. On the other hand, BCM performs similar under different data trace situations.

Figure 7 shows the protocol performance of CoCo and LACE-CoCo. We can see that: (1) LACE-CoCo greatly outperforms CoCo; (2) the improvement is much larger than the improvement in CAOR. The reason is that link correlation in the scenario of multi-message flooding is estimated more accurately. Thus, the desired link correlation can be more accurately identified. Since there are multiple packets to transmit, the *α* is tuned large, containing a more long-term result, which is more stable than the short-term result and is more error-tolerate. (3) The improvements in both channels are similar. The reason is that in CoCo, all network nodes should eventually receive the broadcast packets. Thus, all network neighborhoods can be effective during the transmission progress, and there are more useful routing decisions. While in CAOR, only the nodes on a certain path are used, and there is more performance variation in OR than in multi-message flooding.

## 5. Conclusions

In this paper, we investigated the state-of-the-art approaches of link correlation measurement and proposed a novel link correlation estimation framework (called LACE). Compared with existing beacon-based approaches (BCM), LACE: (1) directly uses data packet receptions for link correlation calculation; and (2) considers both historical data and the instant SINRs. Therefore, LACE provides more accurate link correlation estimation. Moreover, due to the elimination of beacon messages, LACE is much more lightweight than BCM. Experimental results show that LACE achieves more accurate link correlation estimation and improves the performance of existing correlation-based protocols. The future direction lies in finding a theoretical optimal tradeoff between the historical correlation value and the instant value to meet various requirements from upper-layer protocols.

## Figures and Tables

**Table 1.** Notations.

**Notations**	**Descriptions**
$c_{A,B}^{S}$	The link correlation between S→A and S→B
$ch_{A,B}^{S}$	The link correlation value calculated with the data packet trace
$ci_{A,B}^{S}$	The instant correlation value derived from the PHY information
$ct_{A,B}^{S}(k)$	The link correlation value with kcommon receptions
*b_A_*[*i*]	The *i*-th bit in A's bitmap
*R*(*n_r_* = *k*)	The probability with *k* common packet receptions
$p_{A}^{S}(i)$	Packet reception rate of link S→A
$es_{A}^{S}$	symbol error rate
$ec_{A}^{S}$	chip error rate
